# Whole Exome Sequencing of Adult Indians with Apparently Acquired Aplastic Anaemia: Initial Experience at Tertiary Care Hospital

**DOI:** 10.3390/diseases12090225

**Published:** 2024-09-23

**Authors:** Sudhir Mehta, Krishna Mohan Medicherla, Sandhya Gulati, Nidhi Sharma, Rabia Parveen, Ashwani Kumar Mishra, Sonal Gupta, Prashanth Suravajhala

**Affiliations:** 1 Department of General Medicine, SMS Medical College and Hospital, JLN Marg, Jaipur 302004, India; sandygulati60@gmail.com (S.G.); nidhi11112222@gmail.com (N.S.); 2 Department of Biotechnology and Bioinformatics, Birla Institute of Scientific Research, Jaipur 302011, India; kmohan@bisr.res.in (K.M.M.); gupta.sonal1990@gmail.com (S.G.); 3 Bioclues.org, Hyderabad 501511, India; 4 DNA Xperts, Noida 201301, India; rabiaparveen1212@gmail.com (R.P.); ashwani@dnaxperts.com (A.K.M.); 5 Amrita School of Biotechnology, Amrita Vishwa Vidyapeetham, Clappana P.O. 690525, India

**Keywords:** aplastic anaemia, next generation sequencing, systems genomics, exome sequencing

## Abstract

Aplastic anaemia (AA) is a rare hypocellular bone marrow disease with a large number of mutations in the telomerase reverse transcriptase gene (TERT), leading to bone marrow failure. We used our benchmarked whole exome sequencing (WES) pipeline to identify variants in adult Indian subjects with apparently acquired AA. For 36 affected individuals, we sequenced coding regions to a mean coverage of 100× and a sufficient depth was achieved. Downstream validation and filtering to call mutations in patients treated with Cyclosporin A (CsA) identified variants associated with AA. We report four mutations across the genes associated with the AA, *TERT* and *CYP3A5*, in addition to other genes, viz., *IFNG*, *PIGA*, *NBS*/*NBN*, and *MPL*. We demonstrate the application of WES to discover the variants associated with CsA responders and non-responders in an Indian cohort.

## 1. Introduction

Aplastic anaemia (AA) is a rare hypocellular bone marrow disease with bone marrow containing very few hematopoietic cells [1]. Nearly 10–30% patients with apparently acquired AA have mutations in the telomerase reverse transcriptase gene (*TERT*), leading to bone marrow failure [2]. The *TERT* gene is known for maintaining the telomerase ribonucleoprotein complex and plays a crucial role in its regulation, which otherwise causes short telomeres leading to AA. Currently the treatment options for AA include hematopoietic stem cell transplantation (HSCT), anti-thymocyte globulin (ATG), and cyclosporine A (CsA) administered as a standard mode of efficacy. HSCT is the treatment of choice for AA patients with TERT mutations; however, many of them respond to Androgens. In view of the costs associated with HSCT and ATG therapies, many patients are prescribed CsA-Androgen therapy combination. CsA along with Danazol, an anabolic steroid, are administered for treating AA, and they have been assessed in various populations [3,4,5]. When these patients do not respond to immunosuppressive therapy (standard of care), bone marrow transplantation (BMT) is the treatment of choice for AA with documented telomerase mutations [6,7,8,9,10,11].

Over the years, genetic characterization associated with AA has steadily progressed through studies using whole exome sequencing (WES) and mutational screening assays [12,13]. Recently, Zhang et al. have identified potential pathogenic genes for severe AA (SAA) and explored the possible genetic variants in CD8+ T cells [14]. In addition, unlike WES, the evaluation frequency of targeted next-generation sequencing (NGS) in capturing variations with lower allelic burden could be detected with higher rate as the somatic mutation frequency ranges between 5–70% across the NGS studies [15,16,17]. However, the occurrence of such mutations corresponds with the duration of disease, suggesting selective pressure favouring cell survival [18]. Recent efforts have paved the way for identification of AA variants and the likelihood of the effects they cause; for example, the association between homozygous MPL mutations and familial AA is well regarded [19,20]. Intense research over the years has paved the way for diagnosis and treatment of bone therapies, including allogeneic bone marrow transplantation for AA [21,22]. Furthermore, alloimmunization and overall survival are associated in patients with SAA, and recently, granulocyte transfusions have been helpful towards developing a treatment that can bridge patients to curative treatment with HSCT [23]. On the other hand, treatment of newly diagnosed SAA in children has been studied based on evidence-based recommendations [24,25], and this evidence shows high variability after SARS-CoV-2 infection or vaccination [26]. Various drugs, such as eltrombopag, have been checked for efficacy alongside immunosuppressive therapy combined with eltrombopag for the treatment of SAA [27,28]. Cyclosporine plus eltrombopag have been shown to have a similar response and less side effects compared to standard immunosuppressive therapy [29]. There are intended guidelines for the diagnosis and management of SAA and adult AA, which have been in use [30]. Although studies on the magnitude of the problem have elucidated morphological changes associated with AA determinants, there are no published data on *TERT* mutations in Indian patients with acquired AA [31]. Our earlier pilot study was an attempt to identify *TERT* mutations with apparently acquired AA, but the knowledge of using the NGS approaches had not been translated from Telomere dysfunction in the Indian cohort [32]. This could be because of the divergent choice of treatment rendered in those individuals. Therefore, measurement of telomere length and identifying inherent candidate variants would be interesting to understand the disease condition. In this study, we sought to underpin the candidate genetic variants associated with AA from the Indian population using the WES approach. The majority of these patients were of very low socioeconomic status, but because we enrolled transfusion-independent CsA responders, all patients in our cohort were treated with CsA and Danazol. The samples were analysed for variants predicted to be associated with/causal, and further downstream annotation yielded *bona fide* variants with a marked impact on the risk of AA.

## 2. Materials and Methods

**Patients and samples**: The AA subjects were recruited from the department of general medicine, SMS Medical College and Hospital, Jaipur during 2016–’2021, with ethics approval from the institutional ethics committee and Indian Council of Medical Research (ICMR)/Department of Health Research (DHR), Government of India, New Delhi. An informed consent was obtained from all the patients after fully explaining to them about the study and process. The patients’ 2 mL blood sample was drawn through the peripheral vein for WES. A total of 36 AA subject samples with a mean age of 32 have been sequenced, with four samples excluded because the patients were not available for follow-up. Those subjects who responded to CsA and Danazol treatment were considered as CsA responders, while those who underwent blood transfusion were non-responders (Supplementary Table S1). Treatment duration ranged from 6 months to 1 year for follow-up cases; samples for which data were not available due to insufficient follow-up were not included in the final annotations.

**Exome capture and sequencing**: The WES was performed using the QIAamp^®^ DNA Blood Mini Kit (Thermo Fisher Scientific, Waltham, MA, USA) (Cat No: 51104). The quality check (QC) of the genomic DNA (gDNA) was performed using a Qubit^®^ 2.0 Fluorometer followed by agarose gel electrophoresis. Briefly, we used 200 ng of gDNA to generate 300 bp to 350 bp fragments and performed end repair, adapter ligation, and amplification, and adapter ligated DNA was hybridized using the Agilent V5 +  UTRs chemistry (Human All Exon 75 Mb kit) with paired end reads (150 × 2), and an approximate 100× depth of coverage was obtained, resulting in 6–8 GB of data per sample.

**Quality control (QC) and variant calling**: All raw data were analysed in a local 1 TB RAM/64 processors server using our in-house pipeline described previously [33]. The samples were checked for GC bias and duplication levels using FastQC, following which the human genome reference (hg38) was used to align through bowtie2 [11]. After bowtie2 mapping with the hg38 reference, variant calling was performed using VarScan with mutations counted as heterozygous (“het”) through awk/bash scripts. The VarScan prediction tool was employed to check variants filtered for false discovery rate (FDR). The VarScan somatic command was used with the mpileup option, meeting the minimum coverage of 10x to identify possible somatic variants. In this process, their corresponding genotypes for the samples were checked to infer the somatic status. Further predictions identifying “deleterious” mutations were screened for Sanger validation.

**Telomere length analysis and Validation of SNPs**: To check whether or not the AA subjects have shortened telomere length, we used Scicell’s Absolute Human Telomere Length Quantification qPCR Assay Kit (AHTLQ: Catalog #8918). A single copy reference (SCR) primer set was used to amplify a 100 bp-long region on human chromosome 17 and served as a reference for data normalization. The primer sets were validated by qPCR and the gel electrophoresis was run to check the amplification efficiency. All SNPs were mapped to the publicly available databases and the variant effect predictor (VEP) Ensembl suite was used to detect the SNPs that have the minor allele frequency (MAF) cutoff of 0.05. Based on the WES data, the shortlisted variants were cross-checked, primarily using ClinVar, and followed by other validation databases, viz., Varsome, CADD, and GERP scores. The variants were validated by Sanger sequencing and visualized using the Integrated Genome Viewer (IGV) browser for internal cross checking of the variants.

**Statistical analyses and population stratification**: All statistical tests were performed with the sample relationship checks maintained. The bcf/vcf files with a MAF ≤ 0.05 and a minimum DP ≥ 5 were used for mapping the pathogenic variants. First, the mean number of heterozygous variants was calculated, then VerifyBamID was used to infer whether reads were contaminated between samples (*p*-value ≤ 0.05) (*p*-value ≤ 0.05) [34].

## 3. Results and Discussions

### TERT and CYP3A5 Were Shown to Be Harbouring Pathogenic Mutations

We investigated heterozygous variant calls with low-coverage SNPs/indel sites and further explored the singleton mutations with the MAF cutoff of 0.05. We observed the variants associated with a major difference in functional alleles when compared to unaffected samples. From our cohort, two genes, viz., *TERT* (Samples 30, 38 and 50) and *CYP3A5* (sample 44), were shown to be harbouring pathogenic mutations (Table 1). While IFNG, *PIGA*, and *NBS* mutations were found significantly across all subpopulations, we considered the mutations as pathogenic when reported in ClinVar.

**Effect of variants on CsA response**: All four of the mutations were observed in CsA responders with an overall variant detection rate split between six genes, viz., *TERT*, *IFNG*, *PIGA*, *NBS1*/*NBN*, *MPL*, and *CYP3A5*, which were reported in the samples. Apart from *TERT* and *CYP3A5*, the other four genes were not significantly reported in ClinVar. *CYP3A5* is a gene known for mode of action of immunosuppression for CsA response, and the variant is also known to be reported in ClinVar (https://www.ncbi.nlm.nih.gov/clinvar/variation/226021/, last accessed on 3 September 2024). However, a large number of patients, for example, samples 40 and 51, responded within 6 months of therapy with partial remission (free from blood transfusion dependence) maintained.

We found low-confidence *CYP3A5* (NC_000007.14:g.99672916T > C) to be associated with a splice acceptor variant in sample 44. The rs776746 variant of CYP3A5 is associated with intravenous plasma levels in critically ill paediatric patients, which in our case the patient has been susceptible with the presence of this variant [35]. Although they are less significant and benign, we argue that these observations deserve clinical attention, because they are shown to be associated with CsA response in end stage renal failure [36,37]. The reasons why certain patients are more susceptible to transfusion remain to be elucidated. We attribute this to the poor prognosis of some patients, as it may be associated with somatic mutational load [38,39]. As the mutations were reported in CsA responders, we also found mutations attributing to IFNG’s susceptibility to have efficacy in immuno-suppressive therapy [40,41]. All the four mutations across the *TERT* and *CYP3A5* genes, in addition to other genes, viz. *IFNG*, *PIGA*, and *NBS/NBN*, were validated using Sanger sequencing (see data availability). These advances in genomic analysis have shown the complex spectrum of somatic mutations in AA. While PIGA mutations were frequently detected in AA patients at diagnosis, they are less studied as they have low variant allele frequencies.

Furthermore, our telomerase assay showed distinct patterns of telomere length when compared to the normal individuals (see Supplementary Information). While we found as many as nine samples with a telomere length less than the normal range of 10–12 kb, a set of SNPs that exhibited statistically significant association with AA were also checked. The *TERT*/*TERC* mutations are largely associated with pulmonary fibrosis and/or bone marrow failure, cellular senescence, homologous recombination, and/or Dyskeratosis congenita and are telomere-related and/or autosomal dominant [42].

However, our study has inherent limitations. We could not determine whether disease remission was associated with non-genetic or genetic TERT, as treatment durations varied widely between 2.5 months and 4 years in the follow-up cases. Given the paucity of patients with TERT mutations, telomere shortening in AA may involve multiple mechanisms, including telomere damage, genetic defects, and increased stem cell turnover; multiple mechanisms may be involved and require further validation. Due to a lack of family history and samples, germline mutations could not be examined. We found a number of somatic mutations enriched in these pathways, but our exome capture was not a long-term follow-up of patients, which could be a reason why the mutations were not significant.

## 4. Conclusions

In this study, WES was used to screen for mutations in subjects who responded to CsA and those who did not. We have discovered four gene-related mutations: *TERT*, *CYP3A5*, and several others, including *IFNG*, *PIGA*, *NBS*/*NBN*, and *MPL*. This research is presumably the first to document the pathogenic variants connected to Indian AA samples to assess the effect of CsA responders. Despite the relatively small number of samples, and with the exception of the four samples, identifying the patterns to favour CsA is a challenge. For samples that respond to CsA, there is room for genotyping and clinical testing. Our study further suggested that there is no correlation between telomerase length and pathogenesis associated with AA. Our findings should be interpreted with caution given the multiple limitations of this study, but the results encourage future studies with the aim to check the CsA response and assess the mutational burden with large effect size.

## Figures and Tables

**Table 1:** List of somatic mutations from our cohort associated with aplastic anaemia and matched to ClinVar.

Sample#	Gene	HGVS ID	RS ID
30	*TERT*	NM_198253.3(TERT):c.915G > A (p.Ala305=)	rs2736098
38	*TERT*	NC_000005.10:g.1255405G > A	rs33954691
50	*TERT*	NC_000005.10:g.1255405G > A	rs33954691
44	*CYP3A5*	NC_000007.14:g.99672916T > C	rs776746

## Data Availability

The raw reads are made available through sequence read archive project id at PRJNA780657. All Supplementary files (Sanger validation results) are available at https://zenodo.org/me/requests/c6045ead-2193-41f8-bebc-b23bd70f08ba. Last accessed on 3 September 2024.

## Supplementary Materials

The following supporting information can be downloaded at: https://www.mdpi.com/article/10.3390/diseases12090225/s1, Supplementary Table S1: List of samples associated with CsA response. Supplementary information: Telomerase assay results.
